# Single‐cell transcriptome analysis of the mouse and primate ovaries reveals oocyte‐specific expression patterns of risk genes in ovarian aging

**DOI:** 10.1002/mco2.209

**Published:** 2023-02-16

**Authors:** Ye Wei, Miaochun Xu, Xiaoyu Liu, Shitong Lin, Wendi Pei, Ping Zhou, He Liu, Peng Wu, Yang Yu, Canhui Cao

**Affiliations:** ^1^ Department of Gynecology and Obstetrics Key Laboratory of the Ministry of Education Tongji Hospital Tongji Medical College Huazhong University of Science and Technology Wuhan Hubei China; ^2^ Department of Gynecology and Obstetrics Union Hospital Tongji Medical College Huazhong University of Science and Technology Wuhan Hubei China; ^3^ Center for Reproductive Medicine Department of Obstetrics and Gynecology Peking University Third Hospital Beijing China

Dear Editor,

Reproductive longevity is essential for fertility and influences healthy aging in women,^1^ but insights into its underlying biological mechanisms and treatments to preserve it are limited. Since the identification of 290 genetic determinants of ovarian aging via analyzing genetic variations of women in age at natural menopause (ANM), risk loci for ovarian aging have become increasingly clear.^1^ The enrichment of DNA damage response (DDR) processes is an important signature in ovarian aging from the previous Genome‐Wide Association Studies (GWAS) analysis in about 200,000 women of European ancestry, and loss‐of‐function (LOF) variants in these DDR‐associated genes have been demonstrated in mouse female fertility.^1^ However, insights into its expression patterns and differences of risk loci and risk genes in ovaries, especially at single‐cell resolution, are limited. Recently, single‐cell transcriptome data of ovaries from mouse,^2^ young and aged non‐human primates^3^ have been released, which makes it possible to analyze the expression patterns and differences of annotated genes of risk loci between young and old ovaries at single‐cell resolution.

To identify the expression patterns of risk genes (Table S1) in ovarian aging, we explored single‐cell transcriptome data of ovaries from four young (4–5 years old, 1122 cells) and four old (18–20 years old, 1479 cells) cynomolgus monkeys.^3^ After rewiring the risk genes according to the expression of single‐cell transcriptome data in primate ovaries, we found that the expression patterns of risk genes exhibited oocyte‐specific enrichment (Figure 1A), especially the DDR‐associated genes (Figure S1A). More than half of the risk genes were the specific markers of oocytes, including the double‐strand break repair processes (Figure S1B–D). However, only a few risk genes overlapped with marker genes of granulosa cells and showed differential expression in granulosa cells between young and old ovaries (Figure S2A–C). As Wang et al. have clustered the oocytes into four subtypes (C1, C2, C3, and C4) corresponding to oocyte sequential and stepwise subtypes (primordial, primary, secondary, and antral follicles),^3^ we also clustered the oocytes into four subtypes according to their annotations. We then explored the expression levels of risk genes in the four sequential subtypes and found that the risk genes were enriched in antral follicles (Figure 1B). In addition, we found that the expression levels of risk genes gradually increased at different phases of the follicles, from primordial (C1) to antral phase (C4) (Figure 1C). To validate the oocyte‐specific expression patterns of risk genes, we performed the component analysis of the four subtypes using risk genes. Results revealed that the oocytes could be mapped along developmental phases by using risk genes (Figure 1D). Oocyte‐specific expression patterns of risk genes were also validated in single‐cell transcriptome data of the adult mouse ovary, with a slight enrichment in luteal cells (Figure 1E). Furthermore, we tested the oocyte‐specific expression basis by immunofluorescence at different stages of follicles in adult mouse ovaries (Figure 1F). These results suggest that risk genes of ovarian aging show an oocyte‐specific expression pattern at single‐cell resolution of ovaries.

**FIGURE 1:**
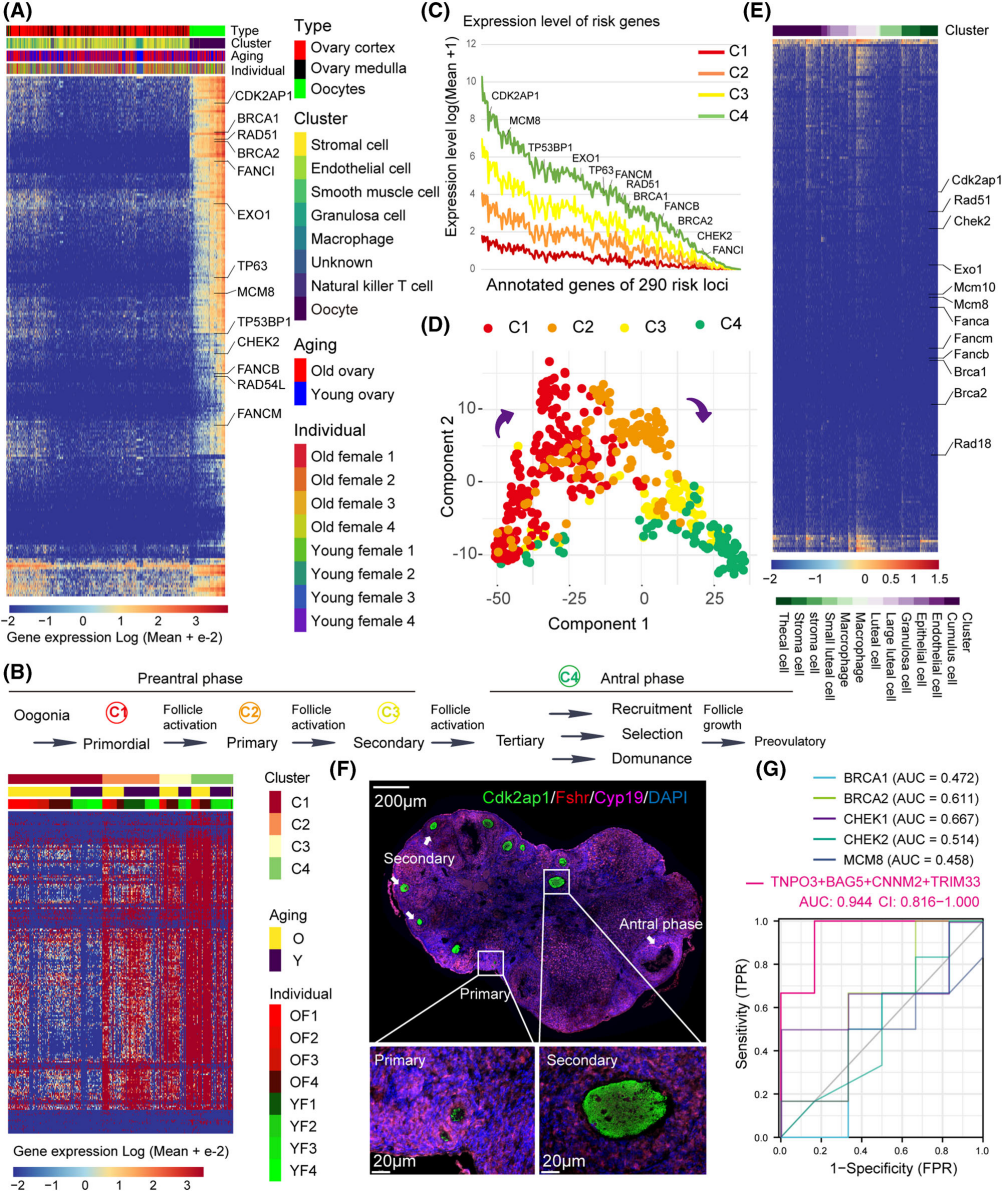
The expression patterns and the characteristics of risk genes in the ovaries. (A) Heatmap of risk genes in the single‐cell transcriptome data of ovaries from four young (4–5 years old, 1122 cells) and four old (18–20 years old, 1479 cells) cynomolgus monkeys, each line represents a gene, and each column represents a cell, and the color annotation is indicated. (B) The oocytes were clustered into four sequential subtypes (C1–C4) that corresponded to oocyte subtypes (primordial, primary, secondary, and antral follicles). Heatmap of risk genes in the single‐cell transcriptome data of oocytes from the young (191 cells) and old (221 cells) cynomolgus monkeys, each line represents a gene, and each column represents a cell, and the color annotation is indicated. O: old ovaries, Y: young ovaries, YF: young female, and OF: old female. (C) Expression level (log [mean + 1]) of risk genes in different sequential subtypes (C1–C4), the *x*‐axis represents annotated genes of 290 risk loci, DDR‐associated genes were indicated. (D) Principal component analysis of the risk genes in different sequential subtypes (C1–C4), each dot represents a cell. (E) Adult mouse ovary (cell = 4363) was downloaded from GSE108097, each line represents a gene, and each column represents a cell, and the color annotation is indicated. (F) Multiple immunohistochemistry staining of risk gene (Cdk2ap1) and granular cell markers (Fshr and Cyp19). Different developmental phases of follicles are indicated. (G) Logistics mode of TNPO3, BAG5, CNNM2, TRIM33, BRCA1, BRCA2, and CHEK2 in GSE155179. *p < 0.05, **p < 0.01, ***p < 0.001

To analyze gene‐expression changes of the risk genes between young and old ovaries during folliculogenesis, we performed differentially expressed genes (DEGs) between young and old oocytes (Figure S3A). Forty‐one upregulated genes and 13 downregulated genes were identified in old oocytes, with the enrichment in the AMPK signaling pathway (hsa04152), DNA synthesis involved in the DNA repair process (GO:0000731), and ovulation cycle process (GO:0042698) (Figure S3B–F; Table S2). Among the DEGs, subcellular locations of TNPO3, BAG5, CNNM2, and TRIM33 were associated with vesicles (Figure S4A; Table S3). The expression patterns of TNPO3, BAG5, CNNM2, and TRIM33 also showed oocyte‐specific enrichment and were significantly high in old oocytes (Figure S4B,C). We then explored the expression signatures of TNPO3, BAG5, CNNM2, and TRIM33 in human cell landscape,^4^ including more than 700,000 cells from more than 50 human tissues, excluding the ovary. We found that the four genes showed slight expression in other tissues and were not classified as specific markers of other tissues (Figure S5A–D), while TNPO3 and CNNM2 were identified as marker genes of oocytes in primate ovaries.^1^ Furthermore, the expression of TNPO3, BAG5, CNNM2, and TRIM33 was identified as a logistics model to predict ovarian aging from other cohorts (GSE155179 and GSE84078), with a higher area under the curve (AUC) than BRCA1, BRCA2 CHEK2, and MCM8 in ovaries (Figure 1G). The AUC of combined logistics model of TNPO3, BAG5, CNNM2, and TRIM33 was higher than BRCA1, BRCA2 CHEK1/2, and/or MCM8 (Figure S5E,F). These results show that the risk genes could be used as a model to predict ovarian aging.

Due to its roles as the oocyte source and the major supplier of steroid sex hormones, the ovary is reputed as a critical female organ in female fertility and women's health and is responsible for human reproduction and endocrine homeostasis.^3^ Ovarian aging is reported to be associated with reproductive aging and ovarian aging events, such as the decrease in the quantity and quality of the oocytes, cycle irregularity and/or menopause, and other disorders.^1^ Moreover, the ovary is observed as one of the organs in humans that shows early‐onset aging‐related dysfunction, with a marked decline after age 30.^3^ As scientists identified 290 risk loci associated with ovarian aging in women, an indepth understanding of the expression patterns of risk genes in ovarian aging is of critical importance.

Although lifetime in developed countries has increased from 45 to 85 years, the age at ANM of women has remained relatively stable, about 50–52 years.^1^, ^3^ A previous study has highlighted the involvement of DNA repair, such as BRCA1/2, in the regulation of ovarian aging and folliculogenesis.^1^, ^5^ LOF alleles in the CHEK2 gene were associated with later ANM, impairment of DDR‐related process led to ovarian aging,^1^ and BRCA1/2 genes were associated with ovarian development and function,^1^, ^5^ The oocyte‐specific expression patterns of risk genes support the molecular roles of these genes in reducing fertility and limiting reproductive lifespan. In our results, AMPK signaling is associated with the aging process or aging‐related disorders, which has been enriched in the DEG cluster of risk genes between the young and old oocytes. The expression of TNPO3, BAG5, CNNM2, and TRIM33 might be good predictors in ovarian aging, which requires more validation data. The molecular roles of risk genes in ovary aging should be validated in the in vivo models, such as conditional knockdown mouse models. In addition, oocyte‐specific expression patterns of risk genes should be validated in monkeys and human ovaries.

Overall, our results presented here demonstrate that oocyte‐specific expression patterns of risk genes in ovarian aging, and unique gene‐expression signatures of risk genes were identified at subtypes of oocytes at sequential developmental phases. In addition, DEGs of risk genes between young and old oocytes were associated with the processes of the ovulation cycle. More importantly, the expression of TNPO3, BAG5, CNNM2, and TRIM33 was identified as a logistics model to predict ovarian aging in oocytes. To our knowledge, this is the first comprehensive analysis of genetic determinants of ovarian aging at single‐cell resolution.

## AUTHOR CONTRIBUTIONS

Canhui Cao, Yang Yu, and Peng Wu developed the concepts. Canhui Cao, Ye Wei, Miaochun Xu, Xiaoyu Liu, and He Liu designed the experiments and performed data analysis. Shitong Lin, Wendi Pei, and Ping Zhou provided feedbacks. Canhui Cao, Yang Yu, and Peng Wu acquired funding. All authors have read and approved the final version of the manuscript.

## CONFLICT OF INTEREST

The authors declare they have no conflicts of interest.

## FUNDING STATEMENT

This work was supported by National Key R&D Program of China (2021YFC2700303, 2021YFC2701201) and National Natural Science Foundation of China (82203453, 82225019, 82192873).

## ETHICS STATEMENT

The animal experiments were approved by the Animal Experiment Ethics Committee of Tongji Hospital and performed according to the AVMA guidelines (TJH‐151‐20210922).

## Supporting information

Supporting Information

Supporting Information

## Data Availability

Single‐cell RNA‐seq data were downloaded from GSE108097 and GSE130664. RNA‐seq data were downloaded from GSE155179 and GSE84078. Any additional information is available from the lead contact upon request.
