# Supplementary material for: Single‐cell transcriptome analysis of the mouse and primate ovaries reveals oocyte‐specific expression patterns of risk genes in ovarian aging
Source: MedComm (2020). 2023 Feb 16;4(2):e209. doi: 10.1002/mco2.209 (PMC9935815; doi:10.1002/mco2.209)
Supplement: Supplementary file 2 — Supporting Information [file MCO2-4-e209-s001.docx]

**Single-cell** **transcriptome analysis of the mouse and primate ovaries reveals oocyte-specific expression patterns of risk genes in** **ovarian ageing**

Ye Wei ^1#^, Miaochun Xu ^1,3#^, Xiaoyu Liu ^1#^, Shitong Lin ^1,3^, Wendi Pei ^2^, Ping Zhou ^2^, He Liu ^1^, Peng Wu ^3*^, Yang Yu ^2*^ & Canhui Cao ^1, 2*.^

^1^ Department of Gynecology and Obstetrics, Key Laboratory of the Ministry of Education, Tongji Hospital, Tongji Medical College, Huazhong University of Science and Technology, Wuhan, Hubei, 430030, China

^2^ Center for Reproductive Medicine, Department of Obstetrics and Gynecology, Peking University Third Hospital, Beijing, 100191, China.

^3^ Department of Gynecology and Obstetrics, Union Hospital, Tongji Medical College, Huazhong University of Science and Technology, Wuhan, Hubei, 430030, China

^#^ These authors contributed equally to the work

^*^ Corresponding author should be addressed to C. C. (canhuicao@foxmail.com), Y. Y. (yuyang5012@hotmail.com) & P. W. (pengwu8626@tjh.tjmu.edu.cn)

**Supplementary information**

**Methods**

**Data acquisition**

Single-cell transcriptomic data of the mouse ovary was downloaded from GSE108097 ^1^, which was generated by Guoji Guo’s Lab, Center for Stem Cell and Regenerative Medicine, Zhejiang University School of Medicine. Single-cell transcriptomic data of non-human primates (*Macaca fascicularis*, 4 young ovaries and 4 aged ovaries) were downloaded from GSE130664 ^2^, which were conducted by STRT-seq protocol. RNA-seq data of oocytes were downloaded from GSE155179 ^3^, which was generated by Shanghai University of Traditional Chinese Medicine. RNA-seq data of mouse ovaries were downloaded from GSE84078 ^4^, which was generated by Burnett School of Biomedical Sciences of Burnett School of Biomedical Sciences.

**Single-cell** **transcriptomic analysis**

The drop-seq core computational tool was used to preprocess of the single-cell transcriptomic data of mouse ovaries. 4,363 cells were processed by Seurat for dimension reduction (tSNE), cell clustering, and differential gene expression analysis via using the digital gene expression data matrix ^1, 5^. Cells were clustered into 14 cell clusters. 2,601 single-cell transcriptomic data of non-human primate ovaries were processed by Seurat for dimension reduction, with the following criteria: 1) log2 (mean expression of the cell cluster (transcripts per kilobase million)) > 0.5; 2) the power value > 0.25; 3) the percentage of such cluster cells (PCT.1) > 0.3. Cells were clustered into 8 types according to the previous study ^2^. Differentially expressed genes (DEGs) of oocyte cells and granulosa cells between young and aged ovaries were analyzed according to the previous study ^2, 6^.

**References**

1. Han X, Wang R, Zhou Y, et al. Mapping the Mouse Cell Atlas by Microwell-Seq. *Cell*. 2018;172(5):1091-1107 e17.

2. Wang S, Zheng Y, Li J, et al. Single-Cell Transcriptomic Atlas of Primate Ovarian Aging. *Cell*. 2020;180(3):585-600 e19.

3. Yuan L, Yin P, Yan H, et al. Single-cell transcriptome analysis of human oocyte ageing. *J Cell Mol Med*. 2021;25(13):6289-303.

4. Schneider A, Matkovich SJ, Saccon T, et al. Ovarian transcriptome associated with reproductive senescence in the long-living Ames dwarf mice. *Mol Cell Endocrinol*. 2017;439:328-336.

5. Satija R, Farrell JA, Gennert D, Schier AF, Regev A. Spatial reconstruction of single-cell gene expression data. *Nat Biotechnol*. 2015;33(5):495-502.

6. Aging Atlas C. Aging Atlas: a multi-omics database for aging biology. *Nucleic Acids Res*. 2021;49(D1):D825-D830.

**Supplementary Figures and Tables**

**
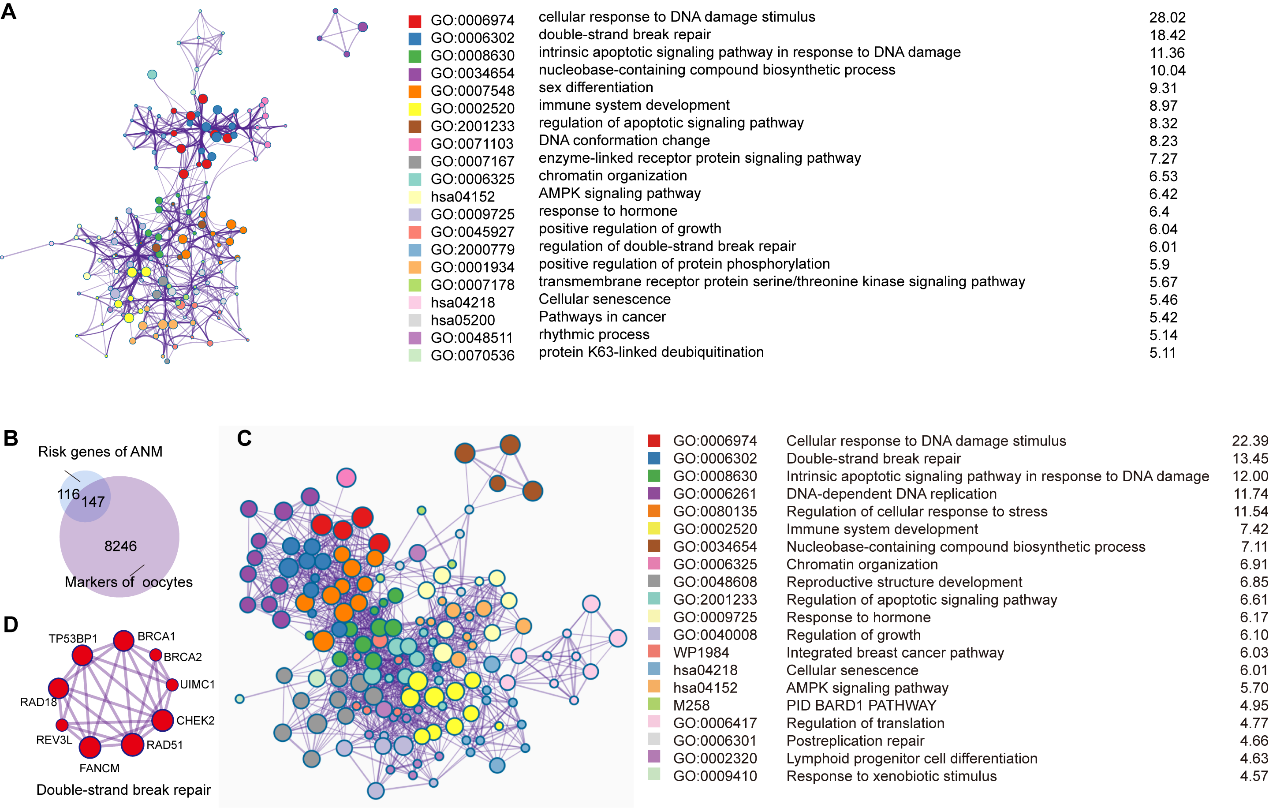
**

**Supplementary Figure 1: Pathway and process enrichment analysis of gene cluster**. (**A**) Pathway and process enrichment analysis of risk genes from the online tool-Metascape (https://metascape.org). (**B**) The Venn diagram between risk genes and markers of oocytes. (**C**) Pathway and process enrichment analysis of 147 genes. (**D**) Protein-protein interaction network of genes related to double-strand break repair (GO:0006302).


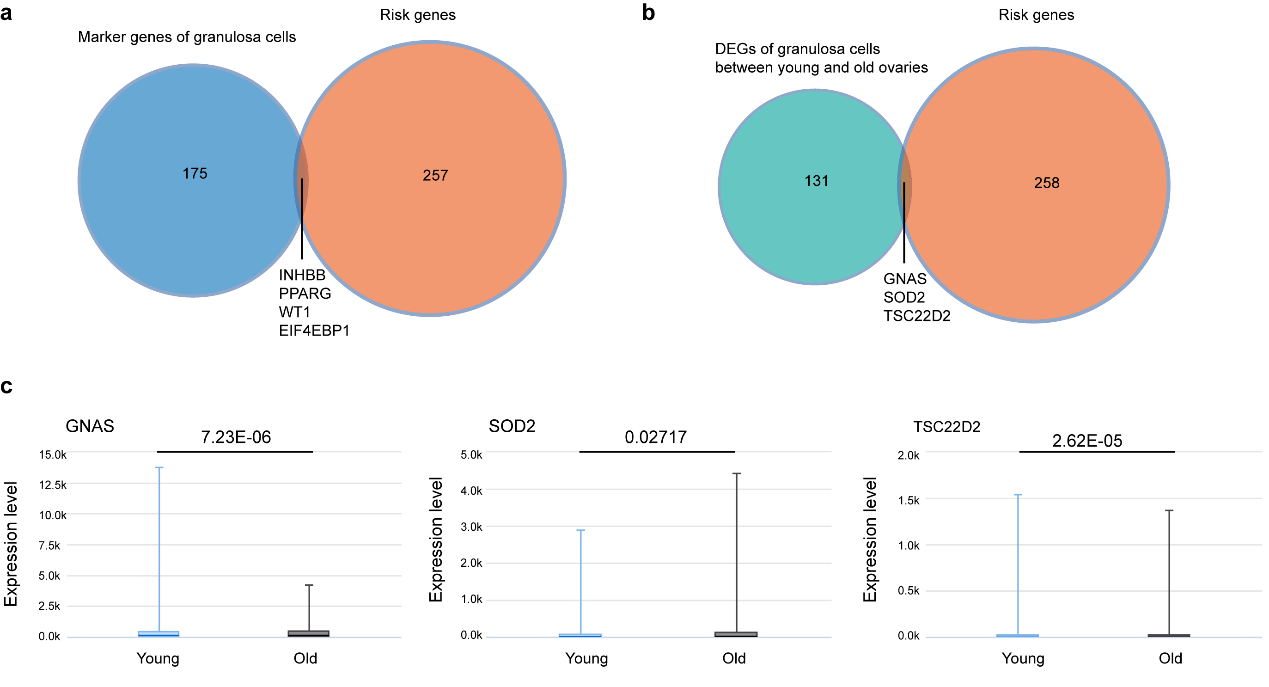


**Supplementary Figure 2: The characteristics of risk genes in granulosa cells.** (**A**) The Venn plot of risk genes and marker genes of granulosa cells. (**B**) The Venn plot of risk genes and DEGs of granulosa cells between young and old ovaries. (**C**) Expression level of GNAS, SOD2, and TSC22D2 in granulosa cells between young and old ovaries.


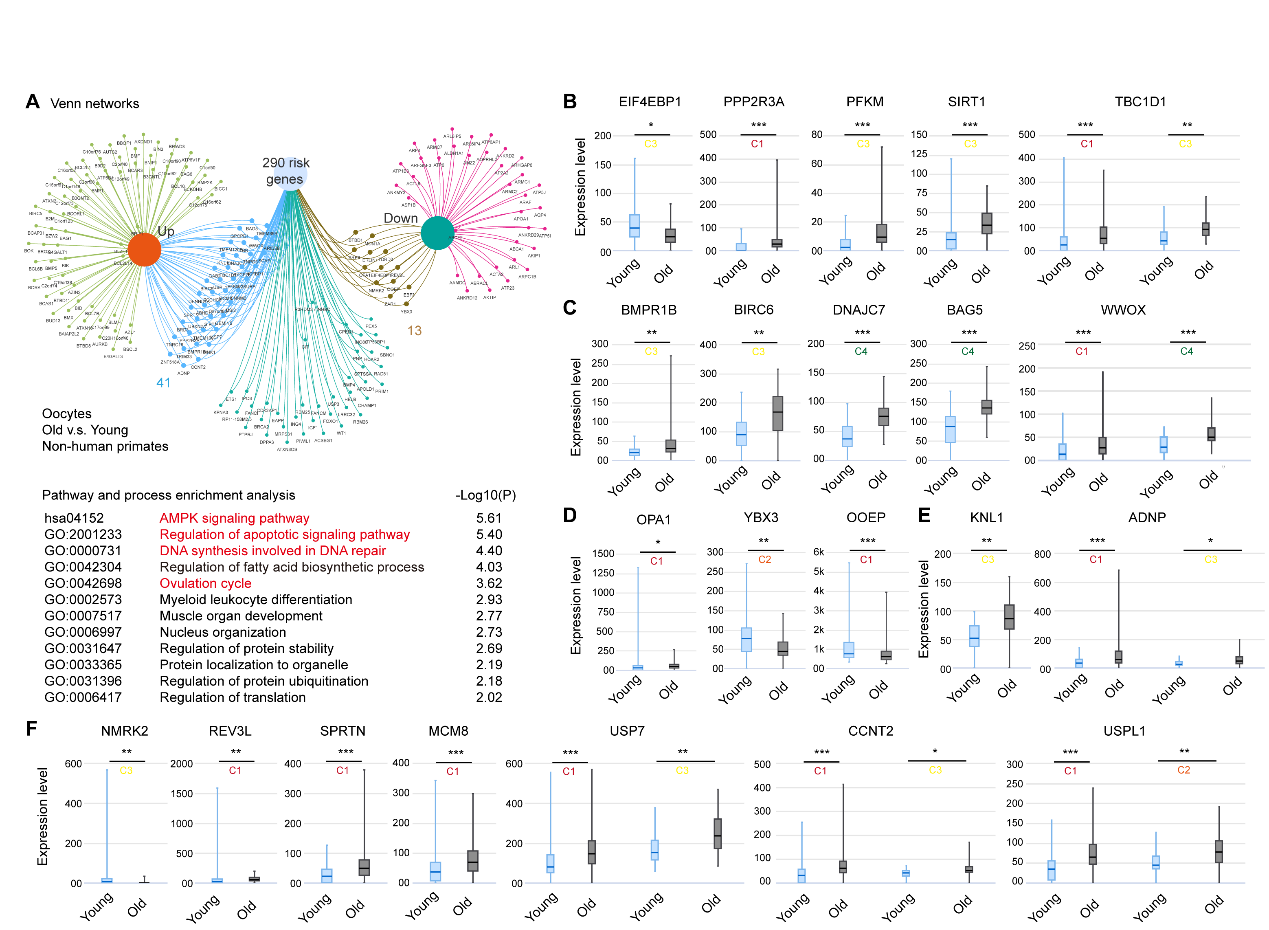


**Supplementary Figure 3: Analysis of overlapped genes between risk genes and DEGs of oocytes.** (**A**) Venn networks between the DEGs (up-regulated genes and down-regulated genes) and risk genes. (**B**) Pathway and process enrichment analysis of overlapped genes between risk genes and DEGs of oocytes, genes related to pathway and process were indicated. Expression levels of genes related to AMPK signaling pathway (**C**), regulation of apoptotic signaling pathway (**D**), DNA synthesis involved in DNA repair (**E**), and ovulation cycle process (**F**) in young and old oocytes. * <0.05, ** <0.01, ***<0.001.


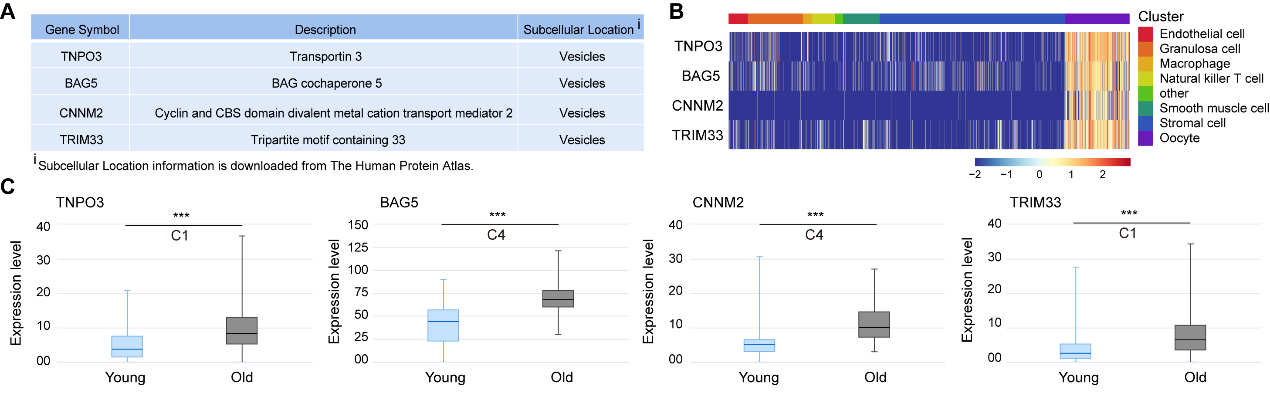


**Supplementary Figure 4: Characteristics of TNPO3, BAG5, CNNM2, and TRIM33.** (**A**) Description and subcellular location of TNPO3, BAG5, CNNM2, and TRIM33. (**B**) Heatmap of TNPO3, BAG5, CNNM2, and TRIM33 in the single-cell transcriptome data of ovaries from four young and four old cynomolgus monkeys, each line represents a gene, and each column represents a cell, the color annotation was indicated. (**C**) Expression levels of TNPO3, BAG5, CNNM2, and TRIM33 in young and old oocytes. * <0.05, ** <0.01, ***<0.001.


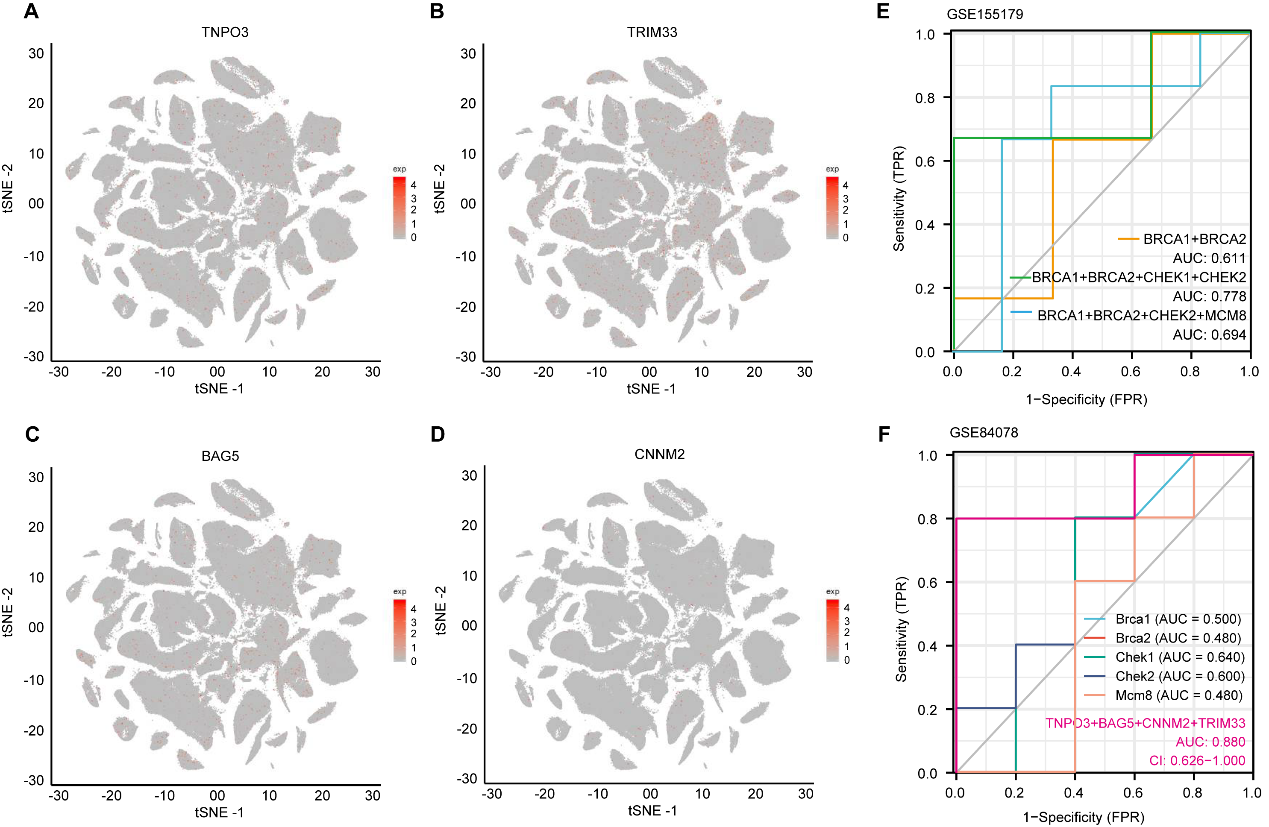


**Supplementary Figure 5: Expression status of TNPO3, BAG5, CNNM2, and TRIM33 and logistics modes.** The expression tSNE map of TNPO3 (**A**), TRIM33 (**B**), BAG5 (**C**), and CNNM2 (**D**). (**E**) The AUC analysis of the combination of BRCA1 and BRCA2, the combination of BRCA1 and BRCA2, MCM8, and CHEK2 in GSE155179. (**F**) The AUC analysis of genes in GSE84078.
